# Optimizing Gingival Biotype Using Subepithelial Connective Tissue Graft: A Case Report and One-Year Followup

**DOI:** 10.1155/2011/263813

**Published:** 2011-07-02

**Authors:** Harpreet Singh Grover, Anil Yadav, Priya Yadav, Prashant Nanda

**Affiliations:** Department of Periodontics and Oral Implantology, SGT Dental College, Hospital & Research Institute, Gurgaon, India

## Abstract

Gingival recession is the exposure of root surfaces due to apical migration of the gingival tissue margins. The principal objectives of treating a gingival recession are to achieve better esthetics and reduce hypersensitivity. The gingival biotype is an important modifying factor in the treatment of gingival recession. The purpose of this paper is to highlight the significance of changing the soft tissue biotype to a more favorable one while attempting root coverage, to achieve more stable and long-lasting results using subepithelial connective tissue graft.

## 1. Introduction

Gingival recession is the exposure of root surfaces due to apical migration of the gingival tissue margins [1]. It may be localized around a single tooth or involves multiple teeth. Sullivan and Atkins [2] classified gingival recession into four categories according to their morphology: (1) deep (more than 3 mm) and wide (larger than 3 mm); (2) shallow and wide; (3) deep and narrow; (4) shallow and narrow. 

Miller [3] in 1985 presented a more elaborate classification of gingival recession according to relation of the marginal tissue to the mucogingival junction and interproximal soft tissue and bone loss. This has been so far the most accepted and widely used classification for gingival recession. 

Gingival recession has a multifactorial etiology associated with periodontal disease, mechanical forces, iatrogenic factors, and anatomical factors [4]. The treatment of the underlying cause is of utmost importance before attempting any root coverage procedure.

The principal objectives of treating a gingival recession are to achieve better esthetics and reduce hypersensitivity [5]. Various treatment modalities have been used to achieve the same including laterally positioned flaps [6], free gingival grafts [7], subepithelial connective tissue grafts [8], coronally advanced flaps [9], guided tissue regeneration [10], and acellular dermal matrix allografts [11].

Among these treatment modalities, variations of subepithelial connective tissue graft procedures have demonstrated the highest success rates with the greatest amount of predictability [12]. The technique was originally described by B. Langer and L. Langer [8] in 1985 and has had several variations in the surgical procedure described since.

More recently a lot of emphasis has also been laid on the soft tissue biotype and its influence as either an etiology or modifying factor leading to recession. Broadly, two extreme tissue biotypes have been described, namely, “thin tissue biotype” characterized by a thin, highly scalloped gingival margin and slender teeth and “thick tissue biotype” characterized by slightly scalloped gingival margin and relatively shorter and wider teeth. A tendency towards greater and more stable soft tissue regain following crown lengthening procedures has been observed around teeth with relatively thicker tissue biotype [13]. 

The purpose of this paper is to highlight the significance of changing the soft tissue biotype to a more favorable one while attempting root coverage, to achieve more stable and long-lasting results using subepithelial connective tissue graft.

## 2. Case Report

**Figure 1:**
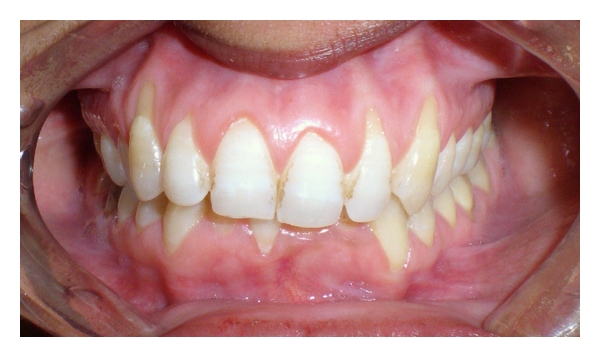
Pretreatment.

**Figure 2:**
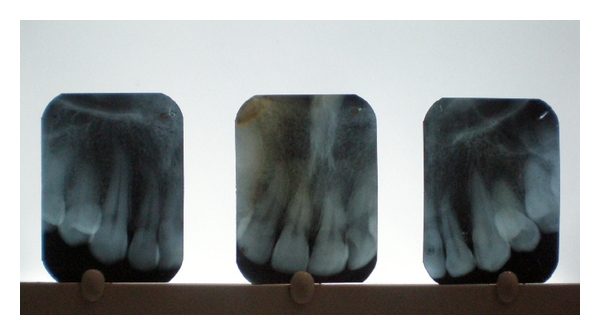
Pretreatment radiographs.

A 25-year-old female patient referred to the Department of Periodontics and Oral Implantology at SGT Dental College, Hospital and Research Institute, Gurgaon with a chief complaint of sensitivity in her upper front teeth. On examination it was seen that the patient had generalized recession particularly in relation to the maxillary anterior teeth from the left to the right maxillary canines (Figure 1). It was diagnosed to be Miller's class I recession with no interdental dental bone loss (Figure 2). The clinical attachment loss was the most severe on both the canines being 4 and 5 mm on right and left maxillary canines, respectively. The soft tissue biotype was examined both visually and by seeing for transparency of a probe inserted through the gingival margin [14]. It was adjudged to be a thin tissue biotype. 

**Figure 3:**
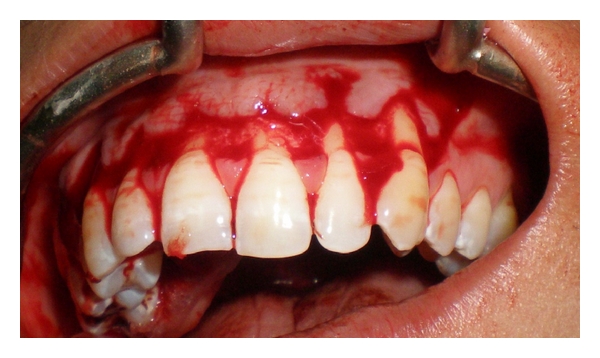
Partial thickness flap elevated.

Thorough scaling and root planning was done for the patient, and she was put on a comprehensive oral hygiene maintenance program. On the day of the surgery, lignocaine 2% was used to anaesthetize the maxillary anterior teeth and the palate bilaterally. Sparing the interdental papilla a partial thickness flap was raised from the left maxillary canine to the right maxillary canine (Figure 3). Some undermining of the tissue was done distal to both maxillary canines, to allow movement of the flap and aid in placement of the subepithelial connective tissue graft.

Due to the amount of connective tissue required to cover the defect, it was preplanned to take the graft bilaterally from the palate. Once the graft was procured, palatal sutures were placed. The connective tissue graft was then trimmed to fit the defect. Following that it was placed to cover the recession defects and sutured in place with Vicryl 4-0 sutures. A periodontal dressing was then placed over the surgical site to aid in uneventful healing.

**Figure 4:**
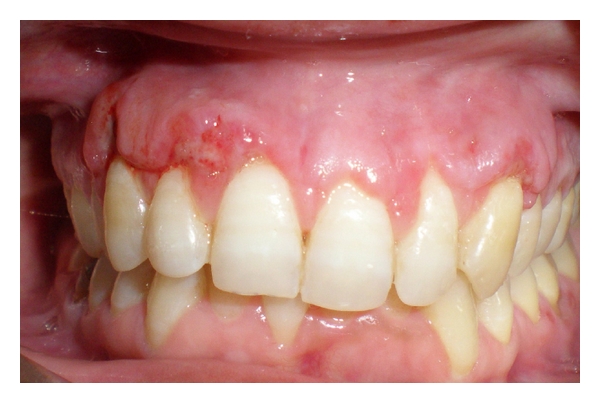
10 days postoperative.

**Figure 5:**
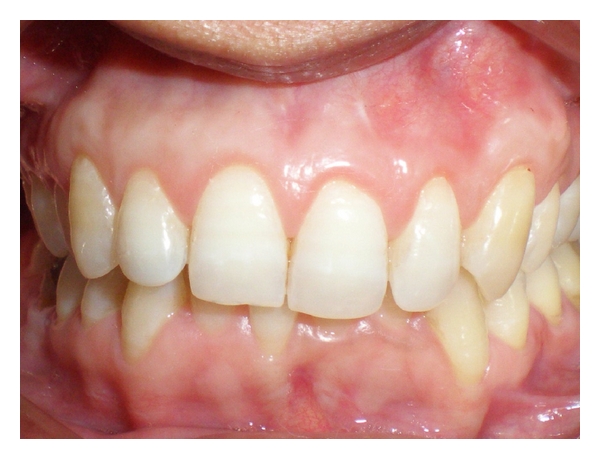
One year postoperative.

Sutures were removed at 10 days postoperatively (Figure 4), and the patient was examined every week for the first one month and every month for the following year. Almost complete root coverage has been achieved in all the teeth which appears to be stable at the end of one year (Figure 5). We were also successfully able to modify the soft tissue biotype into a relatively thicker, more durable one.

## 3. Discussion

The past decade has seen that the goals of periodontal surgery undergo much refinement. Gingival recession associated with hypersensitivity, root caries, and unaesthetic appearance is of frequent concern to both the clinician and the patient. The subepithelial connective tissue graft procedure is the single most effective way to achieve predictable root coverage with a high degree of cosmetic enhancement.

B. Langer and L. Langer [8] initially introduced this technique in 1985 and outlined the indications and procedure for the same. Nelson [15] in 1987 modified it to further increase clinical predictability. This gain in clinical predictability is by use of the bilaminar flap design to ensure graft vascularity (from the bed and the overlying flap) and a high degree of gingival cosmetics from the secondary intention healing of the connective tissue graft.

Wennström [1] in 1996, in a literature review of subepithelial connective tissue procedures, reported an average root coverage of 89% ranging over 50%–98%. This was the highest among all root coverage procedures analysed. Root coverage achieved using the subepithelial connective tissue graft procedure is extremely stable, and thus this procedure is taken as a “Gold Standard” while evaluating the efficacy of other techniques. 

In our particular case we used the Nelson [15] modification of the subepithelial connective tissue grafting technique. The decision to take the connective tissue graft bilaterally from the palate was reached after evaluating the amount of graft required to bring about adequate root coverage. After about 6 months of healing, we were able to achieve almost complete root coverage on all teeth. Complete healing was also achieved at the donor sites.

In clinical practice, the identification of the tissue biotype is essential as variations in it may significantly affect the treatment outcome. More gingival recession has been observed following regenerative procedures in thin tissue biotype, while thick gingiva has been seen to be more resistant to recession following surgery. This may arise due to variability in tissue response to surgical trauma [16]. The role of tissue biotype around dental implants has also been studied extensively, and recession appears to occur more frequently in relation to thin tissue biotype [17].

Thus, the role played by tissue thickness needs to be further elucidated to better understand their influence on treatment planning and outcome.
